# Use of exotic plants to control *Spartina alterniflora* invasion and promote mangrove restoration

**DOI:** 10.1038/srep12980

**Published:** 2015-08-20

**Authors:** Ting Zhou, Shuchao Liu, Zhili Feng, Gang Liu, Qian Gan, Shaolin Peng

**Affiliations:** 1 State Key Laboratory of Biocontrol and Guangdong Key Laboratory of Plant Resources, School of Life Sciences, Sun Yat-sen University, Guangzhou, 510275.

## Abstract

In coastal China, the exotic invasive *Spartina alterniflora* is preventing the establishment of native mangroves. The use of exotic species, control of exotic plant invasion, and restoration of native plant communities are timely research issues. We used exotic *Sonneratia apetala* Buch.-Ham and *S. caseolaris* (L.) Engl. to control invasive *Spartina alterniflora* Loisel through replacement control for five years, which concurrently promoted the restoration of native mangroves. This process includes three stages. I: In a mangrove area invaded by *S. alterniflora*, exotic *S. apetala* and *S. caseolaris* grew rapidly due to their relatively fast-growing character and an allelopathic effect. II: Fast-growing *S. apetala* and *S. caseolaris* eradicate *S. alterniflora* through shading and allelopathy. III: The growth of native mangrove was promoted because exotic plant seedlings cannot regenerate in the understory shade, whereas native mesophytic mangrove plants seedlings can grow; when the area experiences extreme low temperatures in winter or at other times, *S. apetala* dies, and native mangrove species grow to restore the communities. This model has important implications for addressing the worldwide problems of “how to implement the ecological control of invasion using exotic species” and “how to concurrently promote native community restoration during the control of exotic invasion”.

The use of exotic species, control of exotic plant invasion, and restoration of native plant communities are timely research issues. However, they are associated with different research areas and such studies are very difficult to implement successfully. The problem of how to conduct a comprehensive study on these three topics has been undertaken for mangrove restoration in the coastal zone of South China, where the use of exotic *Sonneratia apetala* Buch.-Ham and *S. caseolaris* (L.) Engl. to control invasive *Spartina alterniflora* Loisel and concurrently promote the restoration of native mangroves has been investigated.

The ecological control of invasive species and the restoration of native communities have gained the attention of many researchers^1^,^2^,^3^. The interactions between invaders and competitors may occur directly through competitions for resources or indirectly by allelopathic inhibition^4^. Although certain non-invasive species exhibit stronger competitiveness than invasive species during a particular stage of the life cycle^5^, there are extremely limited successful cases in which noninvasive plants have been used to control invasion^6^,^7^. Thus, sufficiently strong noninvasive competitors and even less invasive exotics may be useful for biological control. A common method is to use a strong competitor for competitive control, thereby contributing to ecological control^6^,^8^. Replacement control is a control method based on the rules of interspecies competition and plant community succession. It uses a more valuable species to naturally replace a harmful plant species^9^ and implement the goal of restoration at a higher level. In general, replacement control adopts native species or plants that have been proven non-harmful to native species after long-term growth. As a competitive plant, the selected species competes with exotic invasive plants and inhibits their growth, usually causing no danger to other useful native plants and also contributing to biodiversity. A few studies have assessed the possibility for the replacement control of invasive plants with native plants. In the Azores community, a native flame tree species has been used to control invasive *Pittosporum undulatum* in a replacement area up to 24% of the invaded area^10^,^11^. It is generally thought that the presence of an exotic plant species is likely to reduce the invasiveness of other invasive plants. In addition, Blank *et al.* (2015)^12^ studied the exotic perennial grass *Agropyron cristatum*, which has been used extensively to control the exotic annual grass *Bromus tectorum* in the intermountain west of the United States, suggesting the co-opting of biological soil space by the perennial grass as another suppressive mechanism. However, the risk of invasion by exotic non-invasive species is unpredictable^13^, and few studies have successfully attempted to use exotic species for competitive replacement control in the field.

Due to their economic attributes, coastal zones are associated with overexploitation, resulting in the shrinkage of contiguous mangrove areas. The degradation and loss of mangrove habitats are most evident in Asia and the Atlantic region. In some places, nearly 70% of the original mangrove habitat has been lost^14^,^15^. The existing natural mangrove area in China is currently only 15,000 hm^2^, although it has historically reached 250,000 hm^2^ 16. Mangrove restoration has become one of the most pressing issues in land improvement in China.

Lost mangrove areas are largely invaded by exotic *S. alterniflora*. Thus, exerting control over *S. alterniflora* is a prerequisite for mangrove restoration. *S. alterniflora* originates from the Atlantic coast. This species presents good performance in tidal-flat preservation, bank protection, wind breaking, and dyke strengthening. *S. alterniflora* was introduced into China in 1979. At present, *S. alterniflora* is the exotic species associated with the most serious invasion in China’s coastal salt marshes^17^,^18^. To date, however, neither physical, chemical, nor biological control through natural-enemy introduction has been able to effectively control *S. alterniflora*^19^,^20^,^21^,^22^. Attempts have been made to “control grass with grass”, i.e., to increase the competitiveness of the native reed (*Phragmites communis* Trin.) by changing the level of resources in the environment and thereby replacing *S. alterniflora*^18^. A previous study^23^ used a native mangrove species (*Kandelia obovata*) for the replacement control of invasive *S. alterniflora*, which also played a certain role in preventing the spread of *S. alterniflora* and restoring the typical complex food web of mature mangrove ecosystems. However, none of the developed methods have been found to be effective for the control of *S. alterniflora*. Therefore, the use of replacement control, particularly the search for effective replacement plants, appears to be of great significance.

Exotic *S. apetala* and *S. caseolaris* have been shown to strongly inhibit *S. alterniflora*^17^,^22^. The present study attempted to use these two exotic mangrove species to control exotic invasive *S. alterniflora* and concurrently promote native mangrove restoration. *S. apetala* was introduced into China from Bangladesh. As an exotic species, *S. apetala* exhibits the characteristics of early successional species, such as heliophytic nutrition, fast growth and reproduction, and a short life cycle^17^. *S. apetala* is commonly used for the ecological restoration of mangroves in South China^18^,^23^,^24^. *S. caseolaris*, originating from Hainan Province, China, represents another exotic species in the study area. Under suitable conditions, their prominent fast-growing characteristics enable *S. caseolaris* and *S. apetala* to rapidly generate a closed canopy and results in forest establishment on bare tidal flats. The establishment of forest will increase soil fertility and improve the habitat, creating favorable environmental conditions for the settlement and growth of other native mangrove plants^25^,^26^. The mixed-species planting of *S. caseolaris* and *S. apetala* can effectively restore mangroves^27^. Additionally, a previous study has shown that *S. apetala* exerts a stronger allelopathic effect than *S. alterniflora*^28^. Because of its relatively low regeneration rate, *S. apetala* presents moderate invasiveness^23^,^24^. Based on these characteristics, *S. apetala* may be useful for the control of invasion by the exotic invasive *S. alterniflora* and for the promotion of the restoration of native mangrove communities.

The present study was designed to address the following research questions: 1) Can exotic species be used to control exotic invasive plants without causing a secondary invasion? 2) Can this ecological control method facilitate the restoration of native plant communities? The replacement model was further proposed, and the underlying mechanism was investigated to provide a paradigm for the comprehensive study of and the development of management practices regarding the use of exotic species to control exotic invasive species and concurrently promote native community restoration.

## Materials and Methods

### Study area description

The study area is located on Qi’ao Island, Zhuhai, Guangdong Province, China (22°23′40′′–22°27′38′′ N, 113°36′40′′–113°39′15′′ E). This area is situated in the Hengmen Estuary in the northeast of Zhuhai and covers an area of 2,400 ha. The area has a vegetation coverage of 85% under a southern subtropical monsoon climate. The mean annual precipitation is 1,964.4 mm, and the mean annual temperature is 22.4 °C. The tide is an irregular semidiurnal tide with a mean high tide level of 0.17 m and a mean low tide level of −0.14 m. The water quality is relatively clean, and the mean annual seawater salinity is 18.2‰. The soil type is coastal saline meadow marsh soil, and the total salt content in the topsoil (0–13 cm) is 20.82‰^29^. This area has been largely invaded and surrounded by *S. alterniflora*^30^.

**Figure 1:**
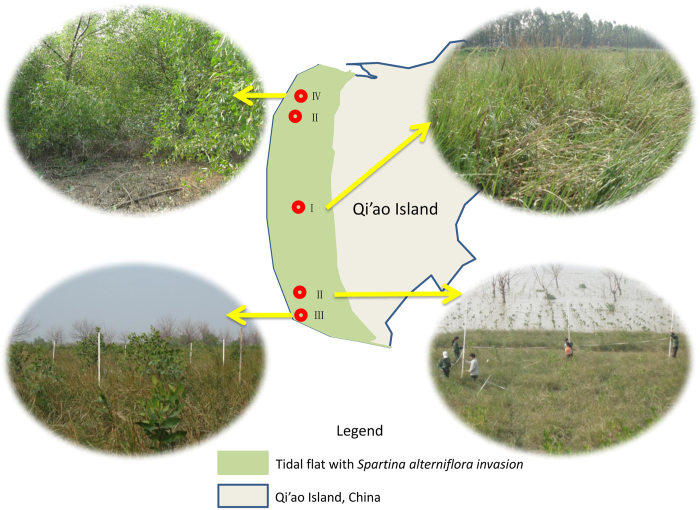
Distribution of the study sites in Qi’ao Island: (I) *S. alterniflora*; (II) *S. apetala + S. alterniflora*; (III) *S. apetala + S. caseolaris* + *S. alterniflora*; and (IV) mature *S. apetala*. Figure 1 was drawn by Ting Zhou, and was generated by Microsoft PowerPoint 2010. The photographs were taken by Jing Li (I and IV) and Yan Zeng (II and III).

The plot chosen was an approximately *~*14.7-ha tidal flat with *S. alterniflora* in the west of Qi’ao Island. In early 2008, *S. apetala* single-species and *S. apetala* + *S. caseolaris* mixed-species plots were planted in the *S. alterniflora* tidal flat (planting density of ~35 trees/100 m^2^, Table 1). Additionally, a pure plot of *S. alterniflora* and a 6–8-year-old mature *S. apetala* plot without *S. alterniflora* growth were selected as controls (Fig. 1).

### Plant survey in sample plots

#### Plot survey

Field sampling was conducted in 2009, 2010, and 2013. Each plot was surveyed to record the height of *S. alterniflora*, the diameter at breast height (DBH) and height of *S. caseolaris* and *S. apetala*, and the understory light intensity. In each plot, a 1-m × 1-m subplot was selected to survey the understory species and record the number and height of the target plants.

### Plant community parameter measurement

#### Plant height and density measurement

The plant height was measured using a diagonal method with 20 plants of *S. alterniflora* selected from each plot. The plant density was surveyed in each plot.

### Light intensity measurement

The understory light intensity was measured along two diagonals of the plot with an electronic illuminometer (BK-1332 A). Measurements were made at 10 points in each plot.

### Biomass estimation and measurement

The biomass of *S. caseolaris* and *S. apetala* was calculated using an empirical formula for fast-growing mangrove plants^31^.


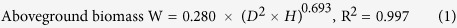


where *D* is the tree DBH (at 1.3-m height), and *H* is the tree height;

*S. alterniflora* biomass measurement: The aboveground biomass of *S. alterniflora* was measured using a harvesting method. Plants were harvested from an area of 1 m × 1 m in each plot. All of the aboveground parts were cut and collected. The fresh plant material was weighed and then dried in an oven at 70 °C to obtain a constant weight.

### Relative growth rate (RGR) calculation



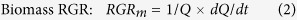



where *Q* is the quantity of the original material, and *dQ/dt* is the transient increment (unit: mg·g^−1^·d^−1^).

### Soil survey in the sample plots

Surface soil (0–15 cm) samples were collected after the plot survey. In each plot, soil samples were collected at nine points in a Z-shaped pattern, and the samples from every three points were pooled to obtain a composite sample by dichotomy. Three composite soil samples were obtained from each plot. The samples were coded and kept in sealable bags.

The soil samples were naturally air-dried, and the plant residues were manually removed. After grinding, the samples were passed through 10- and 100-mesh sieves. The 10-mesh sieved samples were used for pH measurements, and the 100-mesh-sieved samples were used for assays of the soil physicochemical parameters.

The soil pH was measured by water extraction (soil: water = 1: 2.5, w: v) with a pH meter. The soil total carbon and nitrogen contents were determined with a Vario EL Cube elemental analyzer (Elementar, Germany).

### Data analysis

The statistical analyses were performed using SPSS 19.0 (SPSS Inc., Chicago, IL, USA). One-way ANOVA was used to analyze the differences between samples at different times or subjected to different treatments. The least significant difference test was performed to test the significance of the differences. A linear regression analysis was performed to demonstrate the role of declining light intensity in the reduction of *Spartina alterniflora*. The significance level of the differences was set to *P* < 0.05. Plots were generated using Microsoft Excel 2010 and SPSS 19.

## Results

### Influence of different replacement control modes on *S. alterniflora* density

**Figure 2:**
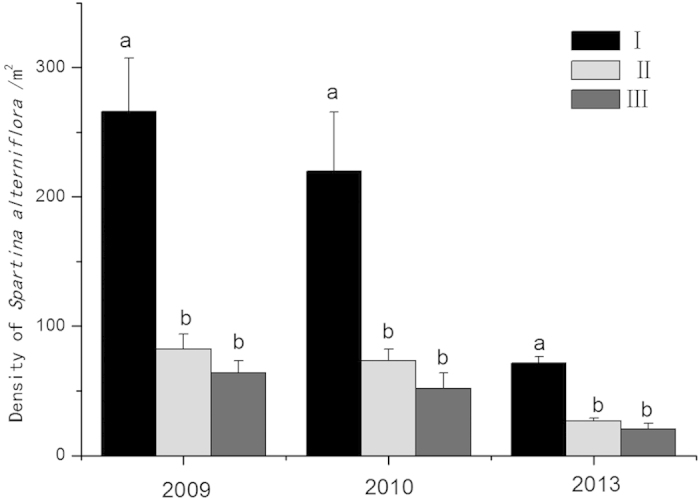
Influence of different replacement control models on *Spartina alterniflora* density (plants/m^2^, mean ± S.E., *p* < 0.05): *(I) *S. alterniflora;* (II) *Sonneratia apetala* controls *S. alterniflora*; and (III) *S. apetala* + *S. caseolaris* control *S. alterniflora*.

After one year of replacement control, the *S. alterniflora* density was 266 plants·m^−2^ in pure plot I compared with 82 and 64 plants·m^−2^ in plots II and III, respectively. The two replacement control measures significantly reduced the density of *S. alterniflora*. Similarly, after two years of replacement control, both the *S. apetala* planting and the *S. apetala* + *S. caseolaris* mixed-species planting showed a significant controlling effect on *S. alterniflora* (*p* < 0. 05). Compared with that in plot I, the *S. alterniflora* density was reduced by 66.82% in plot II and by 76.36% in plot III. After five years of replacement control, the *S. alterniflora* density was 72 plants·m^−2^ in plot I compared with 27 plants·m^−2^ in plot II and 21 plants·m^−2^ in plot III. The *S. alterniflora* density in plot III was reduced by 70.83% compared with that in the plot that was not subjected to replacement control. After five years of control with the single species *S. apetala*, the *S. alterniflora* density decreased by 62.5%. Compared with the pure plot of *S. alterniflora*, plots II and III showed control differences with significant effects (*p* < 0.05; Fig. 2).

### Influence of different replacement control models on *S. alterniflora* biomass

**Figure 3:**
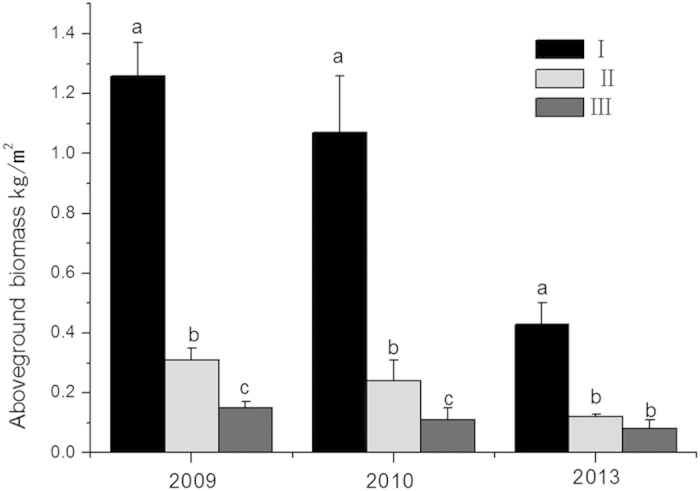
Influence of different replacement control models on *Spartina alterniflora* biomass (kg/m^2^, mean ± S.E., *p* < 0.05): (I) *S. alterniflora*; (II) *Sonneratia apetala* controls *S. alterniflora*; and (III) *S. apetala* + S. caseolaris control *S. alterniflora*.

After two years of replacement control, the aboveground biomass (dry matter) of *S. alterniflora* associated with a mixed-species planting of *S. apetala* + *S. caseolaris* was 0.11 ± 0.04 kg/m^2^, which is significantly less than that associated with a single-species planting of *S. apetala* (0.24 ± 0.07 kg/m^2^). The aboveground biomass of *S. alterniflora* in the pure plot was 1.07 ± 0.19 kg/m^2^. Clearly, the two treatments both resulted in significant reductions in the *S. alterniflora* biomass compared with the control plot. After five years of replacement control, the *S. alterniflora* biomass showed no significant difference between plots II and III. However, the aboveground biomass of *S. alterniflora* had decreased to 0.12 ± 0.01 and 0.08 ± 0.03 kg/m^2^ in the two treatment plots, which are significantly lower than that in the control plot of pure *S. alterniflora* that was not subjected to any replacement control (Fig. 3).

### Differences in the RGR of various plants under different replacement control models

**Figure 4:**
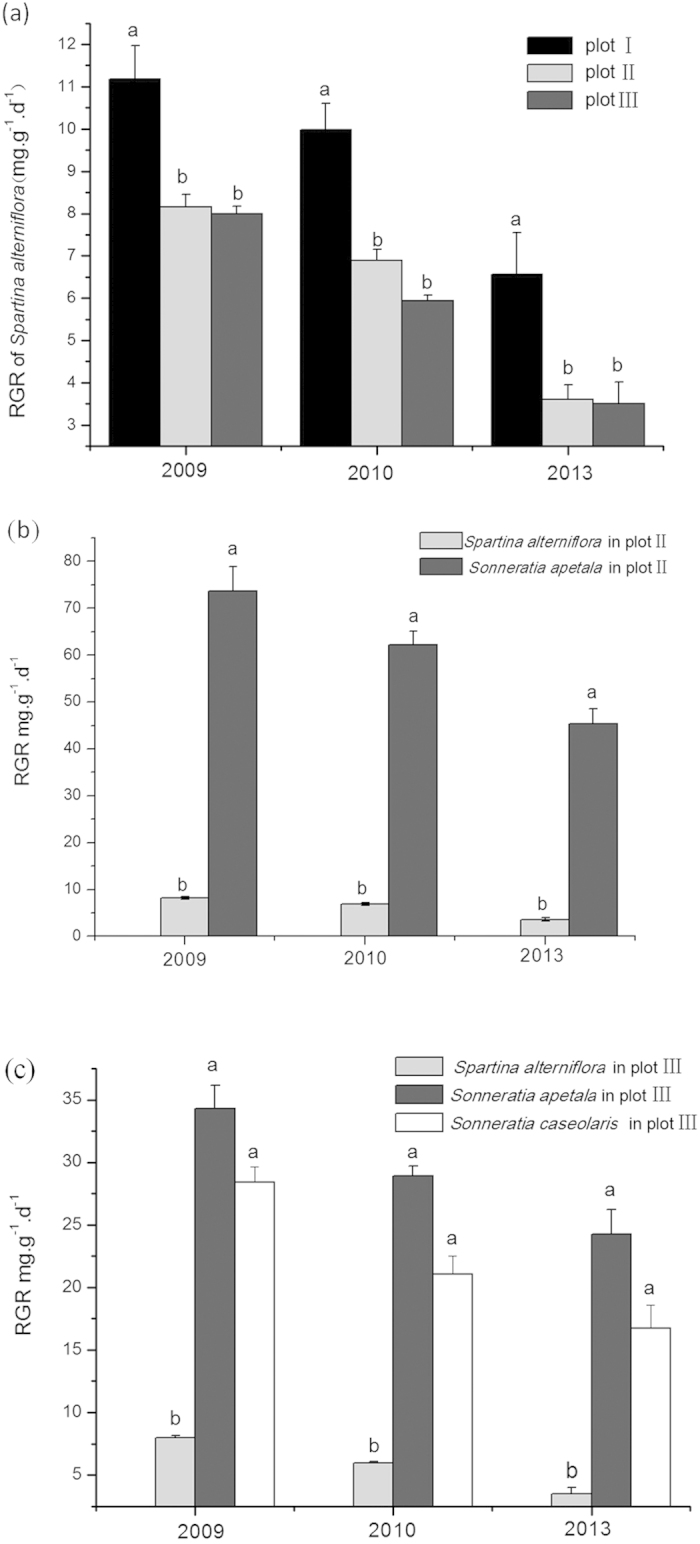
Relative growth rates (RGR) of *Spartina alterniflora*, *Sonneratia apetala*, and *S. caseolaris* in different types of plots. (**a**) One-way ANOVA was used to test for differences in the RGR of *Spartina alterniflora* among plots I, II and III; (**b**) One-way ANOVA was used to test for differences in the RGR between two species in plot II; (**c**) One-way ANOVA was used to test for differences in the RGR among three species in plot III. (mg·g^−1^·d^−1^, mean ± S.E., *p* < 0.05): (I) *S. alterniflora*; (II) *S. apetala* controls *S. alterniflora*; and (III) *S. apetala* + *S. caseolaris* control *S. alterniflora*.

After one, two, and five years of replacement control, *S. alterniflora* presented significantly lower RGR values in plots II and III than in plot I (Fig. 4a), which indicated that the growth of *S. alterniflora* was inhibited under the control of *S. apetala* and *S. caseolaris.* In plot II, *S. apetala* had a significantly higher RGR than *S. alterniflora* (Fig. 4b), and in plots III, both *S. apetala* and *S. caseolaris* had significantly higher RGR *values* than *S. alterniflora* (Fig. 4c). This finding showed that both *S. apetala* and *S. caseolaris* exhibit stronger competitiveness than invasive *S. alterniflora* under coexisting conditions.

### Differences in the understory light intensity and soil properties under different replacement control models

#### Influence of different replacement control models on the understory light intensity

**Figure 5:**
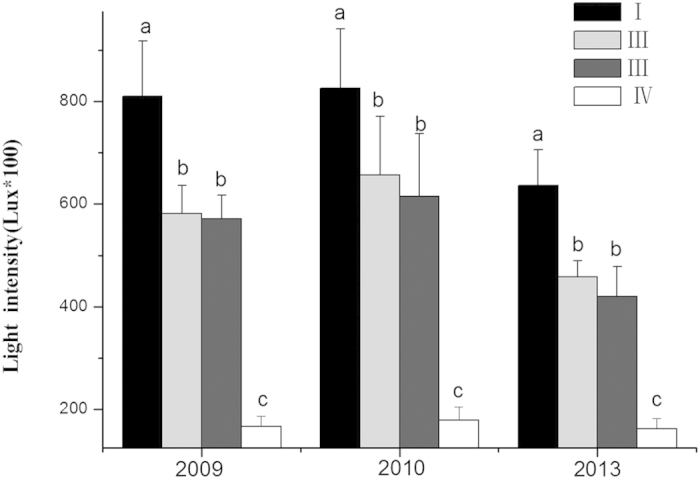
Variations in light intensity under different types of plots (Lux, mean ± S.E., *p* < 0.05): (I) *Spartina alterniflora* plot; (II) *Sonneratia apetala* controls *S. alterniflora* plot; (III) *S. apetala* + *S. caseolaris* control *S. alterniflora* plot; (IV) 6–8-year-old mature *S. apetala* plot.

**Figure 6:**
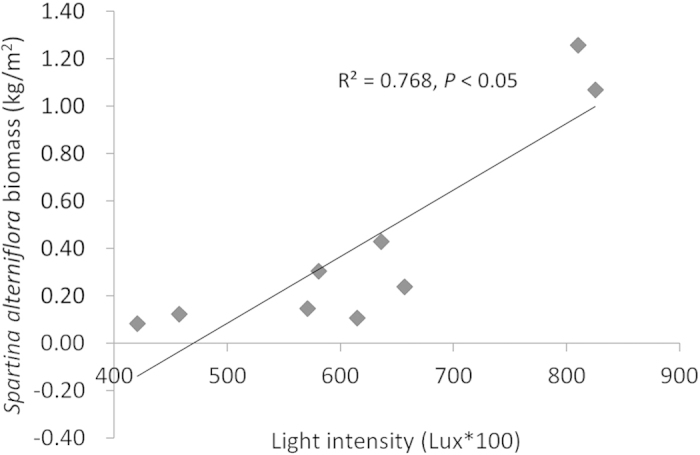
*Spartina alterniflora* biomass decreased with declining light intensity.

The understory light intensity was reduced after five years of replacement control (Fig. 5). In the control plot of pure *S. alterniflora*, the understory light intensity reached 636.00 Lux. In plots II and III, the understory light intensities were 457.60 and 420.27 Lux, respectively, i.e., 71.94% and 66.08% of that in the *S. alterniflora* tidal flat without afforestation. The reductions in understory light intensity were 28.05% in plot II and 33.8% in plot III, showing significant differences (*p* < 0.05). Among the different plots, the lowest light intensity of 161.2 Lux was found in plot I; this minimal value amounts to 25.34% of that in plot I, showing a significant difference (*p* < 0.05). The regression analysis indicated the declining light intensity is a major factor in the reduction of *S. alterniflora* biomass (Fig. 6).

#### Influence of different replacement control models on soil physicochemical properties

**Figure 7:**
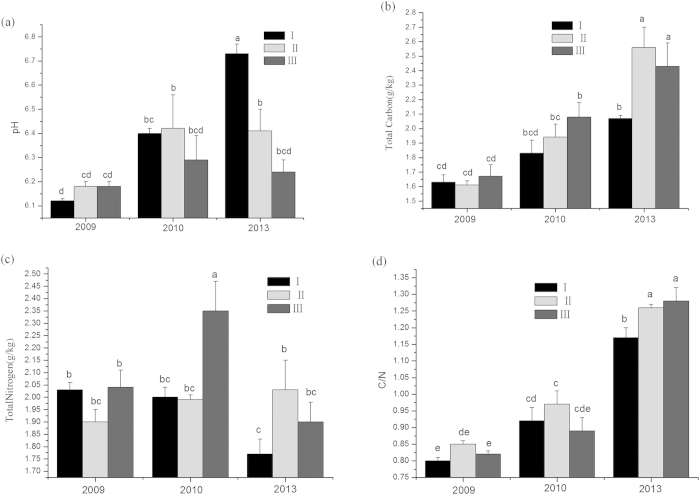
Influence of different replacement control models on soil physicochemical properties: (**a**) pH; (**b**) total carbon; (**c**) total N; (**d**) carbon/nitrogen ratio. (I) *Spartina alterniflora*; (II) *Sonneratia apetala* controls *S. alterniflora*; and (III) *S. apetala* + *S. caseolaris* control *S. alterniflora*.

A comparative analysis of soil physicochemical properties showed that the total carbon content, the total carbon nitrogen and the carbon/nitrogen ratio of the soil increased over time in all three types of plots (Fig. 7). In 2013, the soil total carbon, total nitrogen, and carbon/nitrogen ratio all significantly increased in plots II and III compared with plot I, which indicated that the planting of *S. apetala* and *S. caseolaris* markedly enhanced the nutritional status of the soil in some respects in *S. alterniflora*-invaded areas, resulting in significant improvements in soil properties after five years of replacement control.

#### Restoration of native mangrove plants

**Figure 8:**
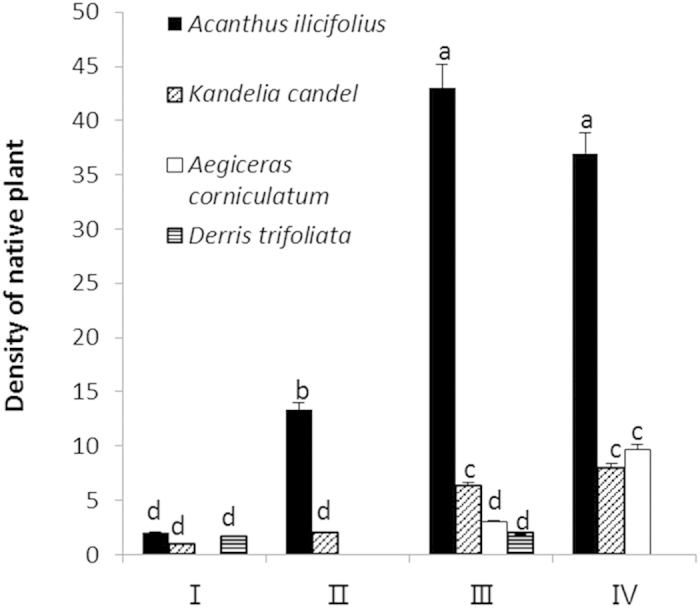
Understory plant density of native mangrove species in different types of plots (plants/m^2^, mean ± S.E.): (I) *Spartina alterniflora*; (II) *Sonneratia apetala* controls *S. alterniflora*; (III) *S. apetala* + *S. caseolaris* control *S. alterniflora*; and (IV) 6–8-year-old mature *S. apetala* plot.

Four native mangrove species, including *Acanthus ilicifolius*, *Kandelia candel*, *Aegiceras corniculatum*, and *Derris trifoliata*, were found in the plots (Fig. 8). In particular, the types and numbers of native mangrove species in plot III were higher than those in plots I and II, indicating that the mixed-species planting of *S. caseolaris* and *S. apetala* can effectively restore native mangrove. In addition, native mangrove plants were found in plot IV, showing that *S. apetala* will not occupy the habitat forever.

## Discussion

### Replacement control model and mechanisms for the control of invasive *S. alterniflora* by exotic *S. apetala* and native mangrove restoration

**Figure 9:**
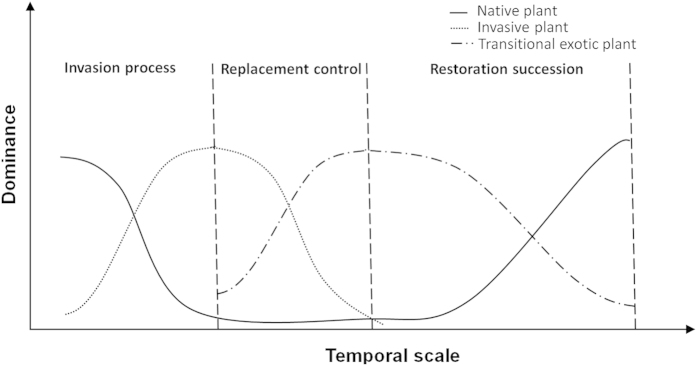
Schematic of the replacement control model for the control of invasive *S. alterniflora* by exotic *S. apetala.* At the stage of exotic species invasion, the native plant dominance gradually declines during invasive plant colonization; after the implementation of replacement control measures, i.e., in the replacement control stage, the invasive plant dominance declines after the planting of a replacement control species. After invasive plant control, the replacement control species can no longer regenerate because of its specific growth characteristics, resulting in the gradual degeneration of the replacement control species and allowing the re-establishment of native plants.

Our study successfully used *S. apetala* and *S. caseolaris* to control *S. alterniflora* (Figs 2 and 3) and concurrently promote the restoration of native mangrove plants (Fig. 8). The entire replacement and restoration model is summarized in Fig. 9. Changes in the dominant species during different stages reflect the model of exotic species control and native community restoration. At the first stage (invasion process), the native plant dominance declines in parallel with exotic species invasion due to interference resulting from human activities. At the second stage (replacement control), the planted transitional exotic species grows fast and thus reduces the dominance of the exotic invasive species. At the following stage (native community restoration succession), the transitional exotic species cannot regenerate and gradually degenerates; however, the shaded habitat built by the transitional exotic species (Figs 4 and 5) provides favorable conditions for the restoration of native plants, thus improving soil properties (Fig. 7) and restoring native communities.

**Figure 10:**
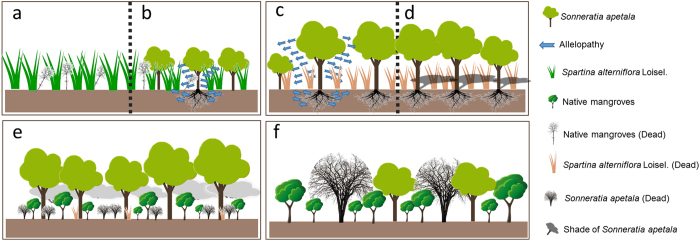
Schematic of the replacement control mechanism. (**a**) The regeneration failure of native mangrove plants and (**b**) the rapid growth of exotic species result in invasion by exotic invasive species and the growth of exotic plants. (**c**) Allelopathy and (**d**) shading result in the replacement of exotic invasive species by exotic plants. (**e**) The shaded environment formed by exotic species promotes the growth of native mangrove seedlings, whereas seedlings of the exotic species are unable to regenerate because of their heliophytic nutrition. (**f**) Exotic species will not cause a secondary invasion due to the failure to regenerate seedlings and the death of tall trees. Figure 10 was drawn by Zhili Feng and Ting Zhou.

The colonization of plants occurs in multiple stages. Because various factors work in different stages, each stage should be studied^32^. In the model of exotic species replacement and native community restoration (Fig. 9), different mechanisms are involved in the various stages (Fig. 10).

Stage I (Fig. 10a,b): Due to the destruction of native mangrove communities, *S. alterniflora* grows rapidly after invasion. The fast-growing characteristic of *S. alterniflora* enables it to rapidly close the canopy and establish a forest to prevent the growth of native mangrove plants. This study planted exotic *S. apetala* in an *S. alterniflora*-invaded area for ecological control. As a pioneer species, *S. apetala* showed a higher growth rate than the invasive species *S. alterniflora* (Fig. 4). Species with higher resource competitiveness and growth rate can overcome their competitors^33^,^34^. For example, a number of invasive species compete with native species due to their high growth rate^34^,^35^,^36^. The ability to grow in *S. alterniflora-*invaded areas is the mechanism of *S. apetala* colonization. Moreover, during the colonization stage of pioneer species, *S. apetala* gains an advantage in its competition with *S. alterniflora* through allelopathy (Fig. 10b). Previous research has shown that *S. apetala* secretes more volatile allelopathic substances than *S. alterniflora*, and the former can also increase the leaf malondialdehyde content and inhibit growth in the latter, thereby achieving the goal of the replacement control of *S. alterniflora*^37^. Species exerting allelopathic effects can obtain more competitive advantages^38^. This mechanism may promote the colonization of *S. apetala* seedlings in high-density communities of *S. alterniflora.* However, a high allelopathic effect suppresses the regeneration of native mangrove plants^39^. This explains why native mangrove plants could not grow in *S. alterniflora* invaded areas.

Stage II (Fig. 10c,d): Once established, on the one hand, an *S. apetala* plot exerts an allelopathic effect to prevent *S. alterniflora* growth, and on the other hand, when the biomass or height of *S. apetala* exceeds that of an invasive species, the closed canopy reduces the understory light intensity (Fig. 5), preventing *S. alterniflora* growth (Fig. 10d). For example, it has been shown that plant communities compete for light resources through asymmetrical competition^40^. Species that win this competition are often higher and can create a shaded habitat for competitors within the community^17^,^41^. Thus, asymmetrical competition between trees (*S. apetala*) and grass (*S. alterniflora*) is conducive to tree growth. Ultimately, the exotic mangrove *S. apetala* replaces the exotic invasive grass *S. alterniflora.*

### Can exotic *S. apetala* cause a secondary invasion?

Once *S. apetala* effectively inhibits *S. alterniflora*, will *S. apetala* undergo large-scale expansion and thus lead to a secondary invasion? The use of exotic species in ecological control and native community restoration may lead to a new invasion—this approach appears to be a paradox. In this context, the regeneration features of moderately invasive species are of particular importance to the restoration of native communities. The present study showed that *S. apetala* substantially and almost completely replaced *S. alterniflora* after five years of control treatment and that native mangrove plants began to grow in the *S. apetala* understory thereafter (Fig. 8). In fact, the growth promotion of late-stage species by this type of plot is fairly common during forest community succession. For example, the tree canopy indirectly promotes the growth of *Quercus suber* seedlings by influencing the herbaceous layer^42^. Thus, “nurse trees” are commonly used in ecological restoration^25^.

In the present study, *S. apetala* seedlings were unable to regenerate because of their inherent growth characteristics. *S. caseolaris* and *S. apetala* are heliophytic fast-growing tree species that can rapidly close the canopy and establish a forest. The resultant low-light conditions inhibit the seed germination and seedling growth of *S. caseolaris*^43^, resulting in a higher mortality of *S. apetala* seedlings. Research shows that with low light resources, *S. apetala* no longer displays its fast-growing characteristic^17^. Consequently, *S. caseolaris* and *S. apetala*, as successional pioneer species, eventually die out naturally. This is another factor limiting the natural regeneration of *S. caseolaris* and *S. apetala*. Additionally, extreme low temperature is the main cause of death for 1-year-old *S. apetala* seedlings. For example, the extreme low temperature over the years on Qi’ao Island is approximately 2.5 °C. On the island, *S. apetala* features a high fruit yield but few perennial seedlings. This is because the seeds have a significantly low natural germination rate in intertidal flats, and seedlings are largely removed due to high tides in winter and autumn^30^. Moreover, the shaded habitat created by *S. apetala* and *S. caseolaris* is conducive to the growth of native mangrove plants (Fig. 10e). Unlike the two exotic species, the native mangrove *A. corniculatum* exhibits strong tolerance to low-light conditions, and its seedlings can grow without being affected by light intensity changes. In shaded environments, *S. apetala* no longer competitively inhibits *A. corniculatum*^17^, allowing seedlings of the native mangrove plants to begin to grow. The low preservation rate of *S. apetala* seedlings and their weak resistance to low temperature indicate that this exotic species will ultimately lose its dominance and be replaced by native mangrove communities.

When invasive plants outcompete native plants, the former often compete with the latter for resources to take over the ecological niche or inhibit the latter through allelopathy. However, *S. apetala* exerts a stronger allelopathic effect on itself than on native mangrove plants, and the autotoxicity to *S. apetala* seed germination is particularly notable^37^. Thus, *S. apetala* is unlikely to harm native mangrove plants through allelopathy. Both the *Sonneratia* species are heliophytes and have no ecological niche overlap with the major native constructive mangrove species^26^. When resources are limited, *S. apetala* competes intensely with *S. caseolaris* but only weakly with native species. Thus, *S. apetala* will not replace native plants and is unlikely to harm native mangrove species. This is the mechanism underlying the fourth stage through which the exotic species ultimately degenerates while native mangrove communities gradually recover to establish forest (Fig. 10f).

### Ecological demonstration of the significance of using exotic species for the control of invasive species in order to address worldwide problems

This study provides an example of the use of exotic species to control invasive species and to further restore native plants. An introductory experiment over 20 years has shown that *S. apetala* grows well in river estuaries in South China; as the pioneer species for artificial mangrove restoration, *S. apetala* has not caused significant ecological invasion^23^. Moreover, mature *S. apetala* forest exhibits strong resistance to cold, i.e., it endures a mean monthly temperature as low as 14.1 °C and an extreme low temperature of 0.2 °C. Presently, *S. apetala* is widespread in the provinces of Hainan, Guangxi, Guangdong, and Fujian, with its northern boundary extending to 28°52′ N^31^. In China, *S. apetala* is distributed in almost all of the latitudinal zones of the native mangrove plant distribution. As long as the introduced area has extreme low temperatures to constrain seedling regeneration, the possibility of secondary invasion can almost be eliminated. Theoretically, the use of *S. apetala* to control *S. alterniflora* can be extended to a relatively large range.

The difference between replacement control and biological control is that the latter directly and specifically kills pests through predators or parasites, whereas the former replaces harmful plants through the natural process of secondary succession, i.e., plant competition in the short term or more complex processes of secondary succession involving a series of plant communities in the long term^9^. Replacement control is a long-term approach that provides ecological benefits, such as soil and water conservation. When conventional biological control methods failed to control *S. alterniflora*, we broke through the conceptual bottleneck to find a strong competitor for *S. alterniflora*, i.e., another invasive species, *S. apetala*, which successfully controls the growth of *S. alterniflora*.

A number of studies have been conducted to “control grass with grass”. However, the majority of the existing research has focused on how to change resources in the environment and use native plants to control invasive plants^44^. For example, a previous study^28^ successfully replaced invasive *Flanueria bidentis* with native foliage. Almost no studies have explored the potential value of invasive plants. Although one study assessed the possibility of using *S. apetala* to control *S. alterniflora*^18^, it involved no long-term field verification. The present study is the first to successfully implement the goal of the “economical, effective, and long-lasting control of *S. alterniflora* with exotic invasive species”. Thus, we believe that “generalized” biological control should be the practice or process through which an undesirable organism is controlled by means of another (beneficial) organism, including the use of not only natural enemies in the narrow sense of biological control but also native species with enhanced competitiveness after environmental resource improvement and the “exotic invasive plants” applied in the present study.

Estuarine wetlands and coastal tidal wetlands are types of ecosystems that provide the highest value of ecosystem services per unit area^45^, but they can be easily invaded^46^. The ecological role provided by mangroves is worth up to $1.6 billion a year. *S. alterniflora* invasion often results in substantial reductions in the distribution area and number of other native populations^47^. How to effectively control *S. alterniflora* and concurrently restore mangrove communities is a worldwide problem requiring attention. Since the late 1970 s, a series of measures have been taken in different regions of the world to mitigate mangrove degradation and loss and to restore mangrove forests^16^. The success rate of the previous measures has been fairly low, and a few conventional control methods are likely to harm native species^48^,^49^.

This study is the first to take advantage of growth competition with an exotic invasive plant (*S. apetala*) for achieving control over *S. alterniflora*. This measure takes full advantage of the ecological characteristics of *S. apetala* and leads to the natural eradication of this species after the completion of its mission, thereby preventing a new invasion. The new approach saves time and money compared with manual control; it avoids pollution and has higher safety than chemical herbicides; additionally, replacement control presents better efficacy, higher safety, and longer-lasting control than conventional biological controls. Even more exciting, during the control process, native plants regain their dominance and are slowly restored. This study provides a new concept for addressing the global problem regarding serious *S. alterniflora* invasions and the difficulty of mangrove restoration: learn from enemies to compete with enemies, i.e., take full advantage of the growth competition of exotic plants for the control of *S. alterniflora*. The proposed method has achieved remarkable results and greatly reduces the cost of exotic plant prevention and native plant restoration. More importantly, the restoration of mangrove communities has brought about ecological benefits that cannot be underestimated.

## Additional Information

**How to cite this article**: Zhou, T. *et al.* Use of exotic plants to control *Spartina alterniflora* invasion and promote mangrove restoration. *Sci. Rep.* **5**, 12980; doi: 10.1038/srep12980 (2015).

## Figures and Tables

**Table 1:** Plot set-up.

**Plot**	**I**	**II**	**III**	**IV**
Plant species	*S. alterniflora*	*S. apetala + S.alterniflora*	*S. apetala + S.caseolaris + S.alterniflora*	*S. apetala* maturestand
Area	3 × 10 m × 10 m	3 × 10 m × 10 m	3 × 10 m × 10 m	3 × 10 m × 10 m

Distribution of experimental plots on the Qi’ao Island, Zhuhai, Guangdong Province, China. I. *Spartina alterniflora* pure plot, II. *Sonneratia apetala + S. alterniflora* plot, III. *S. apetala + S. caseolaris + S. alterniflora* plot, and IV. *S. apetala* mature plot.
